# Impact of low‐level pretransplant donor‐specific antibodies on outcomes after kidney transplantation

**DOI:** 10.1002/iid3.504

**Published:** 2021-08-18

**Authors:** Sandesh Parajuli, Natalie M. Bath, Luis Hidalgo, Glen Leverson, Neetika Garg, Robert R. Redfield, Didier A. Mandelbrot

**Affiliations:** ^1^ Division of Nephrology, Department of Medicine University of Wisconsin School of Medicine and Public Health Madison Wisconsin USA; ^2^ Department of Surgery University of Wisconsin School of Medicine and Public Health Madison Wisconsin USA; ^3^ Division of Transplantation, Department of Surgery University of Wisconsin School of Medicine and Public Health Madison Wisconsin USA; ^4^ Department of Surgery, Hospital of the University of Pennsylvania Perelman School of Medicine Philadelphia Pennsylvania USA

**Keywords:** processes, transplantation

## Abstract

**Background:**

The effect of low‐level pretransplant donor‐specific antibody (DSA) on kidney transplant outcomes is not well described. The goal of this study was to compare outcomes among patients of varying immunologic risk, based on the level of pretransplant DSA.

**Methods:**

We retrospectively reviewed all adult kidney transplant recipients who had undergone a transplant at our center between January 2013 and May 2017. Patients were grouped as negative DSA (mean fluorescence intensity, [MFI_SUM_ < 100]), low‐level DSA (MFI_SUM_ 100–1000), and positive DSA (MFI_SUM_ > 1000). Rejection, infection, graft, and patient survival were outcomes measured.

**Results:**

Of 952 patients, 82.1% had negative DSA, 10.7% had low‐level DSA, and 7.1% had positive DSA. The positive DSA group had the highest rate of antibody‐mediated rejection (10.3%), followed by low‐level DSA (7.8%) and the negative DSA group (4.5%) (*p* = .034). The rate of BK viremia was highest in the positive DSA group (39.7%), followed by the low‐level group (30.4%) and the negative DSA group (25.6%), (*p* = .025). None of the other outcomes, including graft or patient survival, were different between the groups.

**Conclusion:**

While low‐level DSA should not prevent proceeding with kidney transplantation, it should not be ignored. Future studies are needed to investigate the long‐term effects of varying levels of pre‐transplant DSA on outcomes.

## Graphical abstract

The presence of pretransplant donor‐specific antibody (DSA) represents a significant barrier to kidney transplantation for highly sensitized patients as they are less likely to undergo transplants compared with their nonsensitized counterparts. The goal of this study was to compare outcomes among patients of varying immunologic risk, based on the level of pre‐transplant DSA. The positive DSA group had the highest rate of antibody‐mediated rejection (10.3%), followed by low‐level DSA (7.8%) and the negative DSA group (4.5%) (*p* = .034). Also, the rate of BK viremia was highest in the positive DSA group (39.7%), followed by the low‐level group (30.4%) and the negative DSA group (25.6%), (*p* = .025).

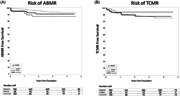

**Abbreviations**
ABMR: antibody‐mediated rejection
ATG: antithymocyte globulin
BMI: body mass index
CIT: cold ischemia time
CMV: cytomegalovirus
cPRA: calculated panel reactive antibodies
DBD: donation after brain death
DGF: delayed graft function
dnDSA: de novo donor‐specific antibody
DSA: donor‐specific antibody
HLA: human leukocyte antigen
KDPI: kidney donor profile index
MFI: mean fluorescence intensity
PRA: panel reactive antibodies
SAB: single antigen bead
TCMR: T‐cell mediated rejection
VXM: virtual crossmatch

## 1 INTRODUCTION

The presence of pretransplant donor‐specific antibody (DSA) represents a significant barrier to kidney transplantation for highly sensitized patients as they are less likely to undergo transplants compared with their nonsensitized counterparts.^1^, ^2^ Luminex single‐antigen bead (SAB) assays are widely used to detect pretransplant DSA, intending to stratify patients by immunologic risk of rejection and other complications.^3^, ^4^ The pretransplant DSA improves the efficiency of organ allocation, as only those patients whose human leukocyte antigen (HLA) antibodies are not donor‐directed or at low/acceptable level will appear on the match run.^5^ However, the level at which DSA becomes clinically significant continues to be widely debated.^3^, ^6^, ^7^ This question remains pertinent since patients who are highly sensitized often undergo complex desensitization protocols, which are associated with other issues including infectious complications, prolonged hospital stay, and increased medical resource utilization.

Previous studies have described the association between pretransplant DSA and increased risk of antibody‐mediated rejection (ABMR); however, there is a lack of data demonstrating at what level of DSA patients are at risk for developing rejection or infectious complications. Therefore, it is critical to better understand the relationship between both low and high‐level pretransplant DSAs and the risk of rejection and infectious complications. Our institution has implemented a pretransplant DSA protocol that stratifies patients based on the intensity of pretransplant DSA.^8^ This protocol divides patients into three groups: (1) negative DSA (absence of pretransplant DSA); (2) low‐level DSA ( ≤1000 mean fluorescence intensity sum [MFI_sum_]); and (3) positive DSA ( >1000 MFI_sum_). The goal of this study is to compare the incidence of rejection, graft survival, patient survival, and infection complications among patients stratified by the DSA level.

## 2 MATERIALS AND METHODS

### 2.1 Data source and patient population

**Figure 1:**
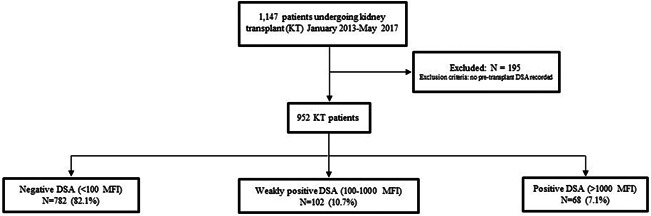
Study flowchart of kidney transplant population. Patients were excluded if DSA was not tested for before transplant. Negative DSA‐MFI < 100, low‐level DSA‐MFI 100–1000, positive DSA‐MFI > 1000. DSA, donor‐specific antibody; KT, kidney transplant; MFI, mean fluorescence intensity

This was a single‐center, longitudinal cohort study of patients undergoing kidney transplantation at our institution between January 2013 and May 2017. Data were obtained from the prospectively collected Wisconsin Allograft Recipient Database and electronic medical records at the University of Wisconsin Hospital. Institutional review board approval was obtained before the study. The activities in this paper conform to the Principles of the Declaration of Istanbul. Patients were excluded if they were not tested for DSA pretransplant or if they were less than 18 years old at the time of transplant. Patients were then grouped according to their pretransplant DSA level as follows: negative DSA (MFI < 100), low‐level DSA (MFI_sum_ 100–1000), and positive DSA (MFI_sum_ > 1000) (Figure 1). In a subgroup analysis, the low‐level DSA group was further divided into two groups, with pretransplant DSA MFI_sum_ less than 500 and MFI_sum_ more than or equal to 500–1000, to compare the incidence of outcomes of interest. Pretransplant DSA levels reported here are from immediate pretransplant serum only and include only DSA present at the time of transplant. The strength of pretransplant DSA was represented as the sum of the mean fluorescence intensity value (MFI_sum_) of all pretransplant DSA, when more than one bead with pretransplant DSA was present. All patients with low‐level DSA had a negative flow crossmatch and those with positive DSA had a negative or low positive flow crossmatch (i.e., median channel shift of <250).

### 2.2 Data collection and outcomes

Primary outcomes of interest included rejection and BK viremia, along with graft survival and patient survival. Graft failure was defined as a return to dialysis, retransplantation, patient death, transplant nephrectomy, or primary nonfunction. All rejection was biopsy‐proven and categorized as ABMR, T‐cell mediated rejection (TCMR), or mixed rejection based on the most recently available Banff criteria.^9^, ^10^ ABMR and TCMR were identified based on kidney biopsy codes that indicated a diagnosis of only ABMR or TCMR, respectively (mixed rejection excluded). Mixed rejection diagnoses were included in the “biopsy‐proven rejection” category along with ABMR and TCMR. Secondary outcomes included the development of de novo DSA (dnDSA), BK viremia cytomegalovirus (CMV) viremia, other viral infection (excluding BK and CMV, but including Esptein‐Barr virus, varicella zoster, and herpes simplex virus), bacterial infection, fungal infection, and delayed graft function. Serum creatinine, estimated glomerular filtration rate (eGFR), and urine protein to creatinine ratio were measured at 1 and 3 years. De novo DSA (dnDSA) was defined as the development of new posttransplant DSA, at any MFI level as previously described,^11^ which was not present pretransplant. CMV infection was defined as any quantifiable viremia via molecular diagnostic testing (positive polymerase chain reaction [PCR]) or biopsy‐proven end‐organ disease via diagnosis code, within the study period. A molecular diagnostic methodology was consistent throughout the study period with the exception of the adoption of the WHO international standard in 2015, which resulted in a conversion from copies/ml to IU/ml. BK viremia was defined as more than 1000 copies/ml. Overall viral infection rates were found using infectious organism codes, which including both CMV and BK virus. Data on organ donors and recipients were collected including ethnicity, gender, age, and BMI (body mass index). Donor type (live, donor after cardiac death, and donor after brain death), and cold ischemia time data were also collected on organ donors. Additional data collected on transplant recipients included the following: induction therapy, MFI_sum_ of pretransplant DSA, blood transfusion, length of pretransplant dialysis, and HLA mismatch.

### 2.3 Immunosuppression

Choice of induction therapy is based on patient‐specific variables including immunological risk, age, primary cause of end‐stage renal disease (ESRD), and plan for early steroid withdrawal. Patients with pretransplant DSA or glomerulonephritis as a cause of ESRD are more likely to receive induction with antithymocyte globulin (ATG). Recipients planned for an early steroid withdrawal are likely to receive alemtuzumab for induction. Alemtuzumab is given as a single intraoperative 30 mg dose for induction. Dosing of ATG for induction at our institution involves an intraoperative dose of 1.5 mg/kg followed by daily postoperative dosing to a goal of 4.5–6 mg/kg based on immunological risk. Basiliximab is given as a single intraoperative 20 mg dose with an optional additional 20 mg dose given on postoperative Day 3, per surgeon discretion. The protocolled posttransplant maintenance immunosuppressive regimen at our center is a triple‐drug regimen consisting of tacrolimus, mycophenolate, and corticosteroids. Institutional protocol dictates tacrolimus troughs range between 5 and 12 ng/ml for the first year after transplant, with higher targets earlier posttransplant.

### 2.4 Desensitization protocol

We have published our desensitization protocol previously.^8^ However, there has been a slight modification since then. During the entire period of the study, we grouped all DSA with MFI_sum_ 100–1000 as a low level and more than 1000 as a positive group. Recipients in the low‐level DSA group receive T‐cell depleting induction with alemtuzumab or ATG, and if a living donor transplant is planned, start tacrolimus 2 mg oral twice daily and mycophenolate 720 mg oral twice daily 1 week before transplant. Recipients in the positive DSA group receive an additional 2 or 3 treatments of plasmapheresis in addition to intravenous immunoglobulin, pre‐and posttransplant, depending upon the types of transplant and feasibility. All patients with pretransplant DSA get close DSA monitoring and protocol biopsy at 3 and 12 months posttransplant, as described before.^12^

### 2.5 Anti‐HLA antibody screening by single antigen bead assay

DSA were detected pre‐ and posttransplant using Luminex SAB (One Lambda) performed according to the manufacturer's instructions with a reduced volume of beads (3 vs. 5 μl).^13^ Our center does not rely on strict MFI cutoffs to assign HLA antibody specificities. Instead, antibodies were identified using multiple criteria including patterns of epitope reactivity, MFI value, specific bead behaviors, and assay background, as described previously.^11^ HLA loci included were HLA‐A, HLA‐B, and HLA‐DR. All DSA detected in this study had MFI values greater than 100. DSAs were classified as de novo if they appeared after transplantation and were not detected in pretransplant samples. Since pretransplant antibodies did not need to meet a minimum MFI threshold to be “identified,” as in studies that use MFI thresholds,^14^ de novo antibody identified in this study is less likely to be due to increases in weak pretransplant DSA.

The strength of dnDSA were represented as the sum of the MFI value of all DSA. Since 2014, routine posttransplant monitoring of DSA was performed on all transplant recipients at 6 and 12 months, and annually thereafter. Patients with a pretransplant cPRA more than 0 were tested at an additional 3‐week time point. In addition, patients with pretransplant DSA were tested at 6 weeks and 3 months posttransplant. Patients with dnDSA underwent transplant biopsy. All patients undergoing renal transplant biopsy for other reasons had DSA testing done as a part of the biopsy visit. The yearly DSA monitoring included patients transplanted before 2014.^15^

### 2.6 Viral monitoring and prophylaxis

At our institution, posttransplant quantitative serum BK PCR is monitored every 2 weeks for the first 3 months, monthly from months 3–12, and at the time of a for‐cause kidney allograft biopsy as described before.^16^ The immunosuppression dose is adjusted for plasma BK PCR more than 1000 copies/ml.

Throughout the study period, CMV prophylaxis protocols at our center were relatively stable. Valganciclovir for CMV prophylaxis or acyclovir for herpes virus prophylaxis were used based on the induction immunosuppression used and risk for infections as described before.^16^

### 2.7 Statistical analysis

Differences between DSA groups were assessed with ANOVA for continuous variables and Fisher's exact tests for nominal variables. The methods of Kaplan–Meier were employed to estimate the incidence of ABMR, ACR, graft survival, patient survival, bacterial infection, viral infection, and fungal infection. Log‐rank test was used to compare rates between DSA groups. Variables with *p* values less than .1 in univariable analyses were included in multivariable analyses. Pretransplant DSA was included in all multivariable analyses regardless of *p* value in univariable analyses. Multivariable analyses were carried out using Cox proportional hazards regression models. *p* values less than .05 were considered to be statistically significant.

## 3 RESULTS

We identified 1147 adult (age ≥ 18 years) patients who underwent kidney transplant from January 2013 to May 2017 at our center. Approximately 195 patients had no available pretransplant DSA testing, so 952 patients were included in our cohort. The majority of patients had no pretransplant DSA (82.1%, *n* = 782; 10.7% (*n* = 102) had low‐level DSA and 7.1% (*n* = 68) had positive DSA (Figure 1). Among 102 recipients with low‐level DSA, 52 had DSA MFI less than 500, and 50 had MFI more than or equal to 500–1000.

### 3.1 Demographics

Demographic data and baseline characteristics for recipients, donors, and graft variables are presented in Tables 1 and 2. The mean posttransplant follow‐up for the cohort was 2.4 ± 1.26 years (median 2.3 years, interquartile range 1.31–3.50). The positive DSA group had significantly younger recipients (*p* < .030) and donors (*p* < .004) compared with the negative and low‐level DSA groups. Overall, the negative DSA group had the lowest rates of previous sensitization events of blood transfusion, previous transplant or pregnancy compared with both low level and positive DSA groups. The majority of patients in both low‐level and positive DSA groups received antithymocyte globulin for induction therapy at 58.8% (*n* = 60) and 67.7% (*n* = 46), respectively (*p* < .0001).

**Table 1:** Characteristics of kidney transplant recipients

Characteristic, recipient (total *N* = 952)	Negative DSA, % (*N* = 782)	Low‐level DSA, % (*N* = 102)	Positive DSA, % (*N* = 68)	*p* value
Age at transplant, mean years	53.3 ± 13.0	51.6 ± 12.0	49.3 ± 12.0	**.030**
Blood transfusion	44.0 (344)	56.9 (58)	64.7 (44)	**.001**
Previous transplant	11.1 (87)	30.4 (31)	51.5 (35)	**<.0001**
Pregnancy	25.2 (197)	47.1 (48)	33.8 (23)	**<.0001**
Induction				**<.0001**
Alemtuzumab	11.1 (87)	8.8 (9)	8.8 (6)	
Basiliximab	66.8 (522)	32.4 (33)	23.5 (16)	
Anti‐thymocyte globulin	22.1 (173)	58.8 (60)	67.7 (46)	
Sum MFI of pre‐transplant DSA, median (interquartile range)	0.0	499.5 (338.5‐696)	1576 (1211‐2239)	**.007**
Class I only		65.6 (67)	52.9 (36)	
Class II only		29.4 (30)	27.9 (19)	
Class I and II		4.9 (5)	19.1 (13)	
Gender				**.0005**
Male	67.0 (524)	48.0 (49)	58.8 (40)	
Race				.773
White	75.3 (589)	70.6 (72)	77.9 (53)	
Black	13.6 (106)	14.7 (15)	11.8 (8)	
Other	11.1 (87)	14.7 (15)	10.3 (7)	
BMI, mean ± *SD*	28.6 ± 5.3	27.9 ± 5.5	27.7 ± 5.3	.254
Pretransplant dialysis, months	26.1	33.6	37.2	.065
HLA mismatch (out of six), mean ± *SD*	3.9 ± 1.5	4.0 ± 1.4	3.7 ± 1.4	.496

Abbreviations: BMI, body mass index; DSA, donor‐specific antibody; HLA, human leukocyte antigen; MFI, mean fluorescence intensity; *SD*, standard deviation.

**Table 2:** Characteristics of kidney donors

Characteristic, donor (total *N* = 952)	Negative DSA, % (*N* = 782)	Low level DSA, % (*N* = 102)	Positive DSA, % (*N* = 68)	*p* value
Gender				.379
Male	54.6 (427)	51.0 (52)	61.8 (42)	
Race				.348
White	90.7 (709)	88.2 (90)	85.3 (58)	
Black	3.3 (26)	2.9 (3)	7.4 (5)	
Other	6.0 (47)	8.8 (9)	7.4 (5)	
Donor type				**.022**
Live	35.9 (281)	22.5 (23)	25.0 (17)	
DBD	44.4 (347)	44.4 (347)	55.9 (38)	
DCD	19.7 (154)	27.5 (28)	19.1 (13)	
Age at donation, mean years	43.9 ± 14.4	41.0 ± 16.6	38.5 ± 14.2	**.004**
BMI, mean ± *SD*	28.1 ± 7.1	28.3 ± 8.2	29.1 ± 8.0	.546
KDPI, % ± *SD*	49.5 ± 26.5	49.3 ± 25.9	40.0 ± 26.1	.051
CIT, mean ± *SD*	14.9 ± 6.7	16.2 ± 6.3	16.1 ± 6.9	.121

Abbreviations: BMI, body mass index; CIT, cold ischemia time; DBD, donation after brain death; DCD, donor after cardiac death; DSA, donor‐specific antibody; KDPI, kidney donor profile index; *SD*, standard deviation.

The negative DSA group had a significantly higher rate of living donors at 35.9% (*n* = 281) compared with both low‐level DSA at 22.5% (*n* = 23) and positive DSA at 25.0% (*n* = 17) (*p* < .022) (Table 2). The positive DSA group trended towards having the lowest KDPI (among deceased donors) although not statistically significant.

### 3.2 Pretransplant DSA

Median MFI in the low‐level DSA group was 500 (interquartile range 339–696) with 66.0% (*n* = 66) of patients having Class I only DSA, 29.0% (*n* = 29) with Class II only and 5.0% (*n* = 5) with both Class I and II DSA. In the positive DSA group, median MFI was 1576 (interquartile range 1211–2239); 52.2% (*n* = 36) had Class I only, 27.5% (*n* = 19) Class II only, and 20.3% (*n* = 14) with both Class I and II DSA.

### 3.3 Rejection, graft, and patient outcomes

**Figure 2:**
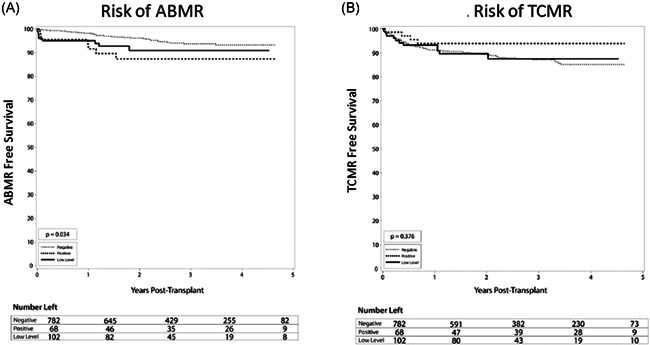
Development of rejection among groups of varying immunologic risk. Kaplan–Meier curves comparing the incidence of rejection between negative, low level, and positive donor‐specific antibody (DSA) groups. (A) Incidence of antibody‐mediated rejection (ABMR). At 1 year, the negative DSA group had 1.7%, low‐level DSA had 4.9%, and positive DSA had 8.3% rate of ABMR (*p* = .033). (B) Incidence of T‐cell mediated rejection (TCMR). At 1 year, the negative DSA group had 8.8%, low‐level DSA had 6.9%, and positive DSA had 6.2% rate of TCMR (*p* = .376)

There was no significant difference in the overall rate of rejections between the groups, with 12.9% in DSA negative group, 13.2% in the low‐level DSA group, and 15.7% in the DSA positive group (*p* = .63). Similarly, in a subgroup analysis, there was no difference in the rate of rejection between low‐level DSA, with 15.4% in DSA MFI 100–500 group compared with 16% (*p* = .99) in the DSA MFI more than or equal to 500–1000 group. Compared with the DSA negative group, ABMR rates were higher in the low‐level DSA group and highest in the DSA positive group. This is demonstrated by Kaplan–Meier analysis (Figure 2A, *p* = .034), and was true at all time points at 1‐year posttransplant and later. At 1 year, ABMR was found in 1.7% of patients with negative DSA, 4.9% with low DSA, and 8.3% with positive DSA (*p* = .033). At the end of the study period, ABMR was found in 4.5% of those with negative DSA, 7.8% with low DSA, and 10.3% with positive DSA (Table 3, *p* = .034). Compared with the negative DSA group, although not statistically significant, those with low‐level pretransplant DSA also had an increased rate of ABMR, 4.5% versus 7.8% (*p* = .09). Similarly, in a subgroup analysis, there was no difference in the rate of ABMR between low‐level DSA, with 7.7% ABMR in DSA MFI 100–500 group compared with 8% in the DSA MFI more than or equal to 500–1000 group (*p* = .96). Also, there was no difference in the rate of ABMR comparing the DSA MFI 100–500 group (7.7%) versus the negative DSA group (4.5%) (*p* = .19) or DSA MFI more than or equal to 500–1000 group (8%) versus the negative DSA group (4.5%) (*p* = .21). In the adjusted analysis, the low‐level DSA group had a two‐fold increased risk of developing ABMR compared with the DSA negative group, but this difference did not reach statistical significance (*p* = .07). Positive DSA patients had a 2.5‐fold increased risk of developing ABMR compared with negative DSA (hazard ratio [HR] 2.5; 95% confidence interval [CI] 1.1–5.8; *p* = .028) (Table 4).

**Table 3:** Comparison of outcomes by groups of varying immunologic risk

Variable	Negative DSA, %	Low level DSA, %	Positive DSA, %	*p* value
Rejection (overall)				
Biopsied‐proven rejection	12.9	15.7	13.2	.638
ABMR	4.5	7.8	10.3	**.034**
TCMR	11.4	10.8	5.9	.376
Graft survival (overall)	95.0	93.1	97.1	.378
Death‐censored graft survival	96.4	94.1	98.5	.209
Patient survival (overall)	97.6	96.1	98.5	.440
Viral infection (excluding BK, CMV)	13.8	13.7	25.0	**.039**
BK viremia (overall)	25.6	30.4	39.7	**.025**
CMV viremia (overall)	31.5	31.4	26.5	.767
Bacterial infection	15.6	20.6	14.7	.428
Fungal infection (overall)	2.0	2.0	5.9	.126
Development of dnDSA (overall)	11.9	17.6	17.6	.078
Delayed graft function	7.3	10.8	14.7	.061
Serum creatinine, mean ± *SD*				
1 year	1.4 ± 0.5	1.4 ± 0.6	1.5 ± 0.6	.837
3 years	1.4 ± 0.6	1.5 ± 0.8	1.4 ± 0.6	.750
eGFR, mean ± *SD*				
1 year	53.3 ± 17.0	51.0 ± 16.2	54.3 ± 21.5	.405
3 years	55.3 ± 18.0	52.3 ± 22.4	54.5 ± 16.4	.738
UPC, mean ± *SD*				
1 year	0.32 ± 0.87	0.38 ± 0.72	0.26 ± 0.25	.75
3 years	0.38 ± 0.76	0.31 ± 0.41	1.1 ± 2.1	**.02**

Abbreviations: ABMR, antibody‐mediated rejection; CMV, cytomegalovirus; DSA, donor‐specific antibody; eGFR, estimated glomerular filtration rate; TCMR, T‐cell mediated rejection; *SD*, standard deviation.

**Table 4:** Risk of antibody‐mediated rejection

Variable	Multivariate analysis	
	HR (95% CI)	*p* value
Pretransplant DSA		
Negative	1	
Low level	2.0 (0.9–4.5)	.070
Positive	2.5 (1.1–5.8)	**.028**
Donor type		
Live	1	
DBD	0.4 (0.2–0.8)	**.006**
DCD	0.6 (0.3–1.3)	.181
Age at transplant (years)	0.98 (0.96–1.0)	.125
Previous blood transfusion		.240
No	1	
Yes	1.2 (0.7–2.0)	

*Note*: Variables with *p* values less than .1 in univariable analyses were included in multivariable analyses.

Abbreviations: CI, confidence interval; DBD, donation after brain death; DCD, donor after cardiac death; DSA, donor‐specific antibody; HR, hazard ratio.

**Figure 3:**
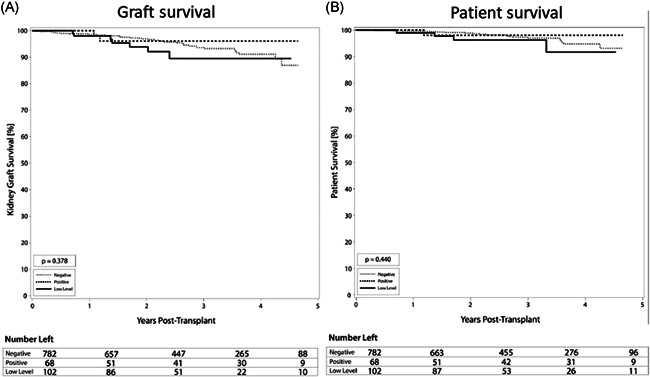
Survival among groups of varying immunologic risk. Kaplan–Meier curves comparing the incidence of kidney graft and patient survival between negative, low level, and positive donor‐specific antibody (DSA) groups. (A) Incidence of kidney graft survival. At 1 year, the negative DSA group had 98.5%, the low‐level DSA group had 97.9%, and the positive DSA group had 100.0% graft survival. (*p* = .378). (B) Incidence of patient survival. At 1 year, the negative DSA group had 99.6%, the low‐level DSA group had 99.0%, and the positive DSA group had 100.0% patient survival (*p* = .440)

There were no statistically significant differences in rates of TCMR, although there was a lower rate in the positive DSA group (5.9%) compared with 10.8% and 11.4% in the low level and negative DSA groups (Figure 2B, *p* = .376). Similarly, in a subgroup analysis, there was no difference in the rate of TCMR between low‐level DSA, with 11.5% TCMR in DSA MFI 100–500 group compared with 10% (*p* = .74) in the DSA MFI more than or equal to 500–1000 group. Also, no significant difference in graft (Figure 3A) or patient (Figure 3B) survival was seen between groups. Overall graft survival remained high in all groups at 97.1% (positive DSA), 93.1% (low‐level DSA), and 95.0% (negative DSA) graft survival (*p* = .38). Similarly, in a subgroup analysis, there was no difference in the rate of graft survival between low‐level DSA, with 94.2% in the DSA MFI 100–500 group compared with 92% in the DSA MFI more than or equal to 500–1000 group (*p* = .73). Additionally, at 1 and 3 years, there were no significant differences in serum creatinine, eGFR, among DSA groups, except urine protein to creatinine ratio was higher in the DSA positive group. Recipient age at transplant and blood transfusion was not associated with an increased risk of ABMR. Induction therapies alemtuzumab and basiliximab were associated with a 2.7‐ and 2.3‐increased risk of TCMR compared with antithymocyte globulin (Table 5), which is notable due to the majority of low level and positive DSA patients receiving antithymocyte globulin for induction.

**Table 5:** Risk of T‐cell mediated rejection

Variable	Multivariate analysis	
	HR (95% CI)	*p* value
Pretransplant DSA		
Negative	1	
Low level	1.2 (0.6–2.3)	.525
Positive	0.7 (0.3–2.0)	.525
Induction group		
Antithymocyte globulin	1	
Alemtuzumab	2.7 (1.3–5.6)	**.006**
Basiliximab	2.3 (1.3–4.0)	**.005**
Donor type		
Live	1	
DBD	0.6 (0.4–0.9)	**.018**
DCD	1.0 (0.6–1.6)	.877
Age at transplant (years)	0.98 (0.97–0.99)	**.012**
Donor age	1.0 (0.99–1.03)	.082
Black race	1.8 (1.1–3.0)	.028

*Note*: Variables with *p* values less than .1 in univariable analyses were included in multivariable analyses.

Abbreviations: CI, confidence interval; DBD, donation after brain death; DCD, donor after cardiac death; DSA, donor‐specific antibody; HR, hazard ratio.

### 3.4 Infectious complications

**Figure 4:**
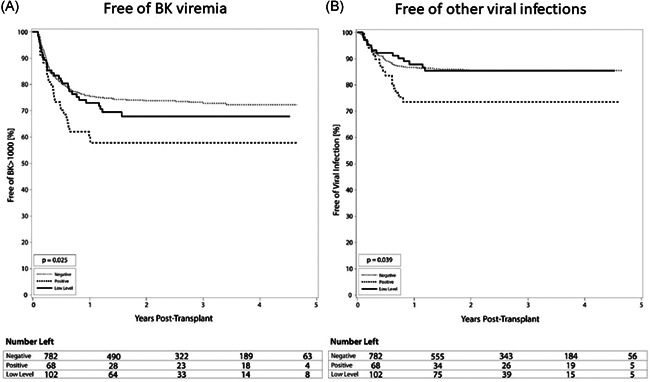
Development of infectious complications among groups of varying immunologic risk. Kaplan‐Meier curves comparing the incidence of infectious complications between negative, low level, and positive donor‐specific antibody (DSA) groups. (A) Incidence of low‐titer BK > 1000 copies/mL. At 1 year, the negative DSA group had 24.4%, the low‐level DSA had 27.0%, and the positive DSA had 39.7% rate of low‐titer BK viremia (*p* = .025). (B) Overall incidence of viral infection. At 1 year, the negative DSA group had 13.4%, the low‐level DSA had 12.2%, and the positive DSA had 26.5% rate of viral infections (*p* = .039)

The rates of BK viremia were roughly correlated with the strength of pre‐transplant DSA MFI, in that the highest rate was found in the positive DSA group (39.7%), intermediate rate in the low‐level DSA group (30.4%), and lowest in the DSA negative group (25.6%) (*p* = .025) (Table 3, Figure 4A). The positive DSA patients were at 1.8‐fold increased risk of developing BK viremia (HR 1.8; 95% CI 1.2–2.6; *p* = .006) (Table 6). Patients in the positive DSA group also developed other infectious complications more frequently than the low‐level DSA and negative DSA groups. Overall, the positive DSA group had the highest rate of viral infections other than CMV and BK viremia at 25.0% with negative and low‐level DSA seeing rates of 13.8% and 13.7%, respectively (*p* = .039) (Table 3, Figure 4B). Additionally, fungal infections were highest overall in the positive DSA group at 5.9% compared to 2.0% in both negative and low‐level DSA groups (*p* = .25).

**Table 6:** Risk of BK viremia

Variable	Multivariate analysis	
	HR (95% CI)	*p* value
Pre‐transplant DSA		
Negative	1	
Low level	1.2 (0.8–1.8)	.289
Positive	1.8 (1.2–2.6)	**.006**
Age at transplant (years)	1.01 (1.002–1.02)	**.021**

*Note*: Variables with *p* values less than .1 in univariable analyses were included in multivariable analyses.

Abbreviations: CI, confidence interval; DSA, donor‐specific antibody; HR, hazard ratio.

## 4 DISCUSSION

Here, we examined kidney allograft outcomes between groups of varying levels of pretransplant DSA. Our results suggest that there is a graded risk of ABMR, such that the higher the pretransplant DSA, the higher the risk of rejection. In our study, patients with low‐level pre‐transplant DSA (MFI_sum_ 100–1000) had an intermediate risk of ABMR (7.8%) compared with rejection rates seen in the negative (4.5%) and positive DSA (MFI_sum _> 1000) (10.3%) groups. Not surprisingly, patients with positive DSA had the highest rate of ABMR. Similarly, the risk of BK viremia was higher in the low level and positive DSA groups, which is likely the result of more aggressive immunosuppression regimens used in these groups, who are thought to be at increased risk of rejection. This association was independent of other risk factors including age at transplant, donor type, and previous blood transfusion. Despite these findings, no significant difference was found between DSA groups for graft or patient survival, presumably reflecting the successful application of more aggressive induction immunosuppression in patients with higher DSA. Also, in a subgroup analysis, there was no difference in the outcomes between patients with low‐level DSA with MFI 100–500 group compared to DSA MFI more than or equal to 500–1000.

It has previously been established that patients with pretransplant DSA are at increased risk of ABMR, graft failure, and chronic rejection.^17^ However, the level at which pretransplant DSA becomes clinically significant has not been well established. Furthermore, it has not been determined if the risk of complications of low‐level DSA (MFI_sum_ 100–1000) more closely resembles negative (MFI_sum _< 100) or positive (MFI_sum_ > 1000) DSA groups, or if the risk is proportional to the strength of DSA. The goal of this study was to determine the relative risk of low‐level pretransplant DSA compared with negative and positive pretransplant DSA.

It is estimated that 30%–40% of potential recipients in the US have pretransplant DSA, which leads to longer wait‐list time and increases the need for desensitization before transplant.^18^ Consequently, these patients receive stronger short‐ and long‐term immunosuppression including higher doses of maintenance therapy.^6^, ^19^, ^20^ Patients with pretransplant DSA are at increased risk of rejection without proper immunosuppression, while at the same time, these patients may also develop infectious complications due to overimmunosuppression. In agreement with this, patients in our cohort with low‐level and positive DSA were at increased risk of developing both ABMR and BK viremia. Importantly, these complications were not associated with inferior patient or graft survival, which may be attributed to early diagnosis and treatment. The weakly positive DSA group had higher incidences of ABMR and BK viremia compared to the negative DSA group, although they did not reach statistical significance in multivariable analyses, likely due to limited power. The potential of crossing pretransplant DSA should be considered within the context of the benefit to patient survival of a kidney transplant across pre‐existing DSA when compared with remaining on the waitlist.^21^

Adebiyi et al.^3^ also investigated the clinical significance of pretransplant DSA in negative flow crossmatched kidney transplant recipients.^3^ Overall, the pretransplant DSA group had a higher incidence of 1‐year acute rejection (ABMR or TCMR) at 15.4% compared with the negative pretransplant DSA group at 11.4%, although not statistically significant. Consistent with our findings, patients with higher levels of pretransplant DSA (MFI_max_ < 1000, 1000–2999, and ≥3000) had higher rates of acute rejection. However, in multivariable analyses, MFI_max_ less than 3000 and negative pretransplant DSA was not associated with significant differences in acute rejection. Regardless of pretransplant DSA status, no difference in 5‐year graft survival was seen. Our findings presented here are not inconsistent with the conclusion presented by Adebiyi et al.,^3^ in that pretransplant DSA should not preclude patients from undergoing transplant; however, our findings suggest that low‐level DSA should not be ignored and that these patients should receive adequate immunosuppression and surveillance.

Although it has been well‐established that pretransplant DSA is associated with increased risk of rejection and inferior graft survival outcomes, limited data exist on the risk of infections in this population. In one study comparing the outcomes among CDC flow crossmatch positive recipients, who had undergone desensitization before transplant, Kim et al.^22^ noted an increased incidence of urinary tract infection, *Pneumocystis jirovecii* pneumonia, and CMV viremia compared with both CDC and flow crossmatch negative recipients. In another study from our institution among 254 kidney transplant recipients, we found pretransplant DSA with MFI more than 500 was associated with an increased risk for the development of BK or CMV infection in the univariate analysis. However, after the adjustment of multiple confounding factors, this association was lost.^19^ The novelty of our study presented here is that both low level and positive pretransplant DSA patients are more likely to develop BK viremia than negative pretransplant DSA patients. Furthermore, positive pretransplant DSA patients continued to be at increased risk of BK viremia in multivariable analyses.

There are several limitations to address in this study. Although the negative DSA group was not associated with significantly higher incidences of graft failure or patient death compared with other groups at 1 year, these could potentially become significantly different over the long term. This study is also limited by the inherent biases associated with retrospective studies. Also, due to the nature of our study, there was some heterogeneity in the recipient's baseline characteristics as the presence of pretransplant DSA is common among recipients with blood transfusion, previous transplants, or pregnancies among females. And per the institutional protocol, depleting agents were chosen for the induction among these recipients. However, to the best of our knowledge, this is the largest series from a single center comparing both rejection and infection outcomes among kidney transplant recipients with varying levels of DSA. Additionally, our single‐center data reflects a more homogeneous clinical approach to patient selection, surgical technique, and medication management, in contrast to registry data involving multiple centers.

In summary, our graft and patient survival outcomes remain quite good regardless of a patient's pre‐transplant DSA status, which is likely the result of our graded approach to induction therapy. While low‐level DSA is not an absolute barrier to transplantation and does not affect graft or patient survival, the increased risk of rejection conferred by low‐level pretransplant DSA should not be ignored. To reduce the risk of rejection, more potent induction immunosuppression may be used in these patients with the caveat that patients may require increased surveillance for infectious complications. Kidney transplant recipients should be aware of these complications before transplant and the alternatives for risk reduction, including paired kidney exchange.

## CONFLICT OF INTERESTS

The authors of this manuscript have no conflict of interests to disclose as described by the Immunity, Inflmmation and Disease.

## AUTHOR CONTRIBUTIONS

**Sandesh Parajuli**: Participated in research design, writing of the article, and performance of the research. **Natalie M. Bath**: Participated in research design, writing of the article, and performance of the research. **Luis Hidalgo**: Participated in research design and writing of the article. **Glen Leverson**: Participated in research design, performance of the research, and data analysis. **Neetika Garg**: Participated in research design, writing of the article, and performance of the research. **Robert R. Redfield III**: Participated in research design and writing of the article. **Didier Mandelbrot**: Participated in research design, writing of the article, and performance of the research.

## Data Availability

The data that support the findings of this study are available on request from the corresponding author. The data are not publicly available due to privacy or ethical restrictions.
